# Supplementary material for: Preventing microalbuminuria with benazepril, valsartan, and benazepril–valsartan combination therapy in diabetic patients with high-normal albuminuria: A prospective, randomized, open-label, blinded endpoint (PROBE) study
Source: PLoS Med. 2021 Jul 14;18(7):e1003691. doi: 10.1371/journal.pmed.1003691 (PMC8279302; doi:10.1371/journal.pmed.1003691)
Supplement: S1 Table — GFR, glomerular filtration rate. (DOCX) [file pmed.1003691.s001.docx]

**Table S1.** Baseline characteristics of patients with glomerular filtration rate measurement by iohexol plasma clearance according to treatment group.

|  | **Benazepril**  *(n=28)* | **Valsartan**  *(n=25)* | | **Combination**  *(n=24)* | |  |
| --- | --- | --- | --- | --- | --- | --- |
| **Demographic characteristics** |  | |  | |  | |
| Age, *years* | 64.8 ± 7.7 | | 64.6 ± 8.6 | | 66.4 ± 8.3 | |
| Male sex, *n (%)* | 24 (85.7) | | 21 (84.0) | | 20 (83.3) | |
| Known duration of diabetes, *years* | 13.6 ± 9.7 | | 12.0 ± 6.8 | | 11.1 ± 5.9 | |
| Smoking status, *n (%)* |  | |  | |  | |
| Never smoked | 8 (28.6) | | 9 (36.0) | | 10 (41.7) | |
| Former smoker | 15 (53.6) | | 14 (56.0) | | 9 (37.5) | |
| Current smoker | 5 (17.8) | | 2 (8.0) | | 5 (20.8) | |
| **Clinical features** |  | |  | |  | |
| BMI, *Kg/m^2^* | 30.6 ± 4.7 | | 29.6 ± 4.7 | | 30.3 ± 3.8 | |
| Systolic blood pressure, *mmHg* | 137.3 ± 12.6 | | 134.8 ± 13.6 | | 139.8 ± 11.2 | |
| Diastolic blood pressure*, mmHg* | 80.5 ± 9.9 | | 81.9 ± 6.3 | | 83.6 ± 7.8 | |
| MAP, *mmHg* | 99.4 ± 9.6 | | 99.6 ± 7.6 | | 102.3 ± 8.1 | |
| **Laboratory parameters** |  | |  | |  | |
| HbA1c, *mmol/mol* | 59.1 ± 12.9 | | 51.3 ± 8.4 | | 53.2 ± 13.4 | |
| HbA1c, *%* | 7.6 ± 1.2 | | 6.8 ± 0.8 | | 7.0 ± 1.2 | |
| Serum glucose, *mg/dL* | 163.4 ± 37.0 | | 155.1 ± 43.5 | | 152.2 ± 42.5 | |
| Serum potassium, *mg/dL* | 4.09 ± 0.39 | | 3.91 ± 0.35 | | 3.90 ± 0.30 | |
| Hemoglobin, *g/dL* | 14.2 ± 1.1 | | 14.2 ± 1.1 | | 13.7 ± 1.0 | |
| Total cholesterol, *mg/dL* | 179.6 ± 34.2 | | 164.5 ± 31.7 | | 169.3 ± 31.5 | |
| HDL cholesterol, *mg/dL* | 45.8 ± 12.2 | | 45.5 ± 11.3 | | 43.7 ± 12.7 | |
| LDL cholesterol, *mg/dL* | 108.2 ± 29.2 | | 98.3 ± 29.1 | | 105.4 ± 31.4 | |
| Triglycerides, *mg/dL* | 143.6 ± 100.7 | | 123.4 ± 59.0 | | 124.5 ± 62.1 | |
| **Kidney function parameters** |  | |  | |  | |
| Serum creatinine, *mg/dL* | 0.94 ± 0.24 | | 0.98 ± 0.23 | | 1.00 ± 0.21 | |
| Measured GFR, *mL/min/1.73 m^2^* | 85.93 ± 14.30 | | 84.68 ± 20.85 | | 83.04 ± 16.76 | |
| Urinary albumin excretion, *µg/min* | 10.64[7.35-13.50] | | 8.59[6.42-11.76] | | 8.06[5.92-12.31] | |
| **Patients with medications, *n (%)*** |  | |  | |  | |
| ***- Antihypertensive agents*** |  | |  | |  | |
| *- Any* | 21 (75.0) | | 20 (80.0) | | 13 (54.2) | |
| *- Diuretics* | 9 (32.1) | | 10 (40.0) | | 4 (16.7) | |
| *- Calcium-channel blockers* | 13 (46.4) | | 11 (44.0) | | 6 (25.0) | |
| *- Beta-blockers* | 9 (32.1) | | 8 (32.0) | | 5 (20.8) | |
| *- Sympatholytic agents* | 0 | | 0 | | 0 | |
| *- ACE inhibitors* | 0 | | 1 (4.0) | | 0 | |
| *- ARB* | 0 | | 0 | | 0 | |
| ***- Lipid-lowering agents:*** |  | |  | |  | |
| *- Any* | 15 (53.6) | | 19 (76.0) | | 14 (58.3) | |
| *- Statins* | 13 (46.4) | | 18 (72.0) | | 12 (50.0) | |
| *- Fibrates* | 2 (7.1) | | 2 (8.0) | | 2 (8.3) | |
| ***- Hypoglycemic agents:*** |  | |  | |  | |
| *- Any* | 27 (96.4) | | 24 (96.0) | | 22 (91.7) | |
| *- Oral hypoglycemic agents* | 24 (85.7) | | 23 (92.0) | | 20 (83.3) | |
| *- Insulin* | 4 (14.3) | | 3 (12.0) | | 6 (25.0) | |
| *- Diet alone* | 1 (3.6) | | 1 (4.0) | | 2 (8.3) | |

Data are mean ± SD, median [IQR] or numbers (percentages). Abbreviations: ACE, Angiotensin converting enzyme; ARB, Angiotensin receptor blocker; BMI, Body-mass index; HbA1c, Glycated hemoglobin; MAP, Mean arterial pressure. Glycated hemoglobin (HbA1c) values were expressed by using percentage (%) units according to the Diabetes Control and Complication Trial (DCCT) and mmol/mol units according to the International Federation of Clinical Chemistry and Laboratory Medicine (IFCC).
