# Supplementary material for: Preventing microalbuminuria with benazepril, valsartan, and benazepril–valsartan combination therapy in diabetic patients with high-normal albuminuria: A prospective, randomized, open-label, blinded endpoint (PROBE) study
Source: PLoS Med. 2021 Jul 14;18(7):e1003691. doi: 10.1371/journal.pmed.1003691 (PMC8279302; doi:10.1371/journal.pmed.1003691)
Supplement: S2 Table — (DOCX) [file pmed.1003691.s002.docx]

**Table S2.** Baseline predictors of microalbuminuria development at univariable and multivariable analyses.

|  | Univariable | | Multivariable | |
| --- | --- | --- | --- | --- |
|  | Beta | P value | Beta* | P value |
| Study treatment, *benazepril vs combination therapy* | -0.0425 | 0.739 |  |  |
| Study treatment, *valsartan vs combination therapy* | 0.1370 | 0.275 |  |  |
| Age, *years* | 0.0274 | 0.025 | 0.0308 | 0.024 |
| Sex, *(M/F)* | 0.2043 | 0.048 |  |  |
| BMI, *Kg/m^2^* | -0.0035 | 0.856 |  |  |
| Smoking status, *(never vs current and former)* | -0.0246 | 0.784 |  |  |
| Systolic blood pressure, *mmHg* | -0·0015 | 0.810 |  |  |
| Diastolic blood pressure*, mmHg* | -0.0077 | 0.487 |  |  |
| Mean Arterial Pressure, *mmHg* | -0.0063 | 0.558 |  |  |
| Known duration of diabetes, *years* | 0.0195 | 0.132 |  |  |
| HbA1c, *mmol/mol* | 0.1455 | 0.019 |  |  |
| Serum creatinine, *mg/dL* | 1.7019 | 0.0002 | 1.4171 | 0.003 |
| Total cholesterol, *mg/dL* | -0.0005 | 0.860 |  |  |
| Triglycerides, *mg/dL* | 0.0033 | 0.005 | 0.0036 | 0.005 |
| Log-urinary albumin excretion, *µg/min* | 1.9402 | <0.0001 | 1.9706 | <0.0001 |

**Beta expressed as standardized coefficients*.
