# Supplementary material for: Preventing microalbuminuria with benazepril, valsartan, and benazepril–valsartan combination therapy in diabetic patients with high-normal albuminuria: A prospective, randomized, open-label, blinded endpoint (PROBE) study
Source: PLoS Med. 2021 Jul 14;18(7):e1003691. doi: 10.1371/journal.pmed.1003691 (PMC8279302; doi:10.1371/journal.pmed.1003691)
Supplement: S3 Table — non-SAE, nonserious adverse event; SOC, system organ classification. (DOCX) [file pmed.1003691.s003.docx]

**Table S3.** Number (%) of patients with at least one non-serious adverse event (non-SAE) according to treatment arm by MedDRA System Organ Classification (SOC).

|  | **Benazepril**  *(n=209)* | **Valsartan**  *(n=201)* | **Combination**  *(n=202)* |
| --- | --- | --- | --- |
| Blood and lymphatic system disorders | 22 (10.5%) | 22 (10.9%) | 23 (11.4%) |
| Cardiac disorders | 51 (24.4%) | 61 (30.3%) | 55 (30.2%) |
| Congenital, familial and genetic disorders | 1 (0.5%) | 3 (1.5%) | 2 (1.0%) |
| Endocrine disorders | 2 (1.0%) | 2 (1.0%) | 2 (1.0%) |
| Ear and labyrinth disorders | 2 (1.0%) | 2 (1.0%) | 6 (3.0%) |
| Eye disorders | 28 (13.4%) | 26 (12.9%) | 28 (13.9%) |
| Gastrointestinal disorders | 33 (15.8%) | 35 (17.4%) | 39 (19.3%) |
| General disorders and administration site conditions | 34 (16.3%) | 35 (17.4%) | 33 (16.3%) |
| Hepatobiliary disorders | 16 (7.7 %) | 9 (4.5%) | 11 (5.4%) |
| Immune system disorders | 5 (2.4%) | 1 (0.5%) | 0 (0.0%) |
| Infections and infestations | 51 (24.4%) | 56 (27.9%) | 48 (23.8%) |
| Injury, poisoning and procedural complications | 14 (6.7%) | 10 (5.0%) | 18 (8.9%) |
| Investigations | 35 (16.7%) | 27 (13.4%) | 28 (13.9%) |
| Metabolism and nutrition disorders | 47 (22.5%) | 55 (27.4%) | 53 (26.2%) |
| Musculoskeletal and connective tissue disorders | 42 (20.1%) | 40 (19.9%) | 44 (21.8%) |
| Neoplasms benign, malignant and unspecified (including cysts and polyps) | 5 (2.4%) | 5 (2.5%) | 3 (1.5%) |
| Nervous system disorders | 36 (17.2%) | 27 (13.4%) | 34 (17.8%) |
| Psychiatric disorders | 10 (4.8%) | 4 (2.0%) | 8 (4.0%) |
| Renal and urinary disorders | 17 (8.1%) | 18 (8.9%) | 23 (11.4%) |
| Reproductive system and breast disorders | 21 (10.0%) | 21 (10.4%) | 12 (5.9%) |
| Respiratory, thoracic and mediastinal disorders | 21 (10.0%) | 13 (6.5%) | 25 (12.4%) |
| Skin and subcutaneous tissue disorders | 14 (6.7%) | 7 (3.5%) | 10 (4.9%) |
| Surgical and medical procedures | 3 (1.4%) | 0 (0.0%) | 1 (0.5%) |
| Vascular disorders | 19 (9.1%) | 31 (15.4%) | 38 (18.8%)* |

Chi-square test; **P*<0.01 versus Benazepril.
