# Supplementary figures and images for: Preventing microalbuminuria with benazepril, valsartan, and benazepril–valsartan combination therapy in diabetic patients with high-normal albuminuria: A prospective, randomized, open-label, blinded endpoint (PROBE) study
Source: PLoS Med. 2021 Jul 14;18(7):e1003691. doi: 10.1371/journal.pmed.1003691 (PMC8279302; doi:10.1371/journal.pmed.1003691)

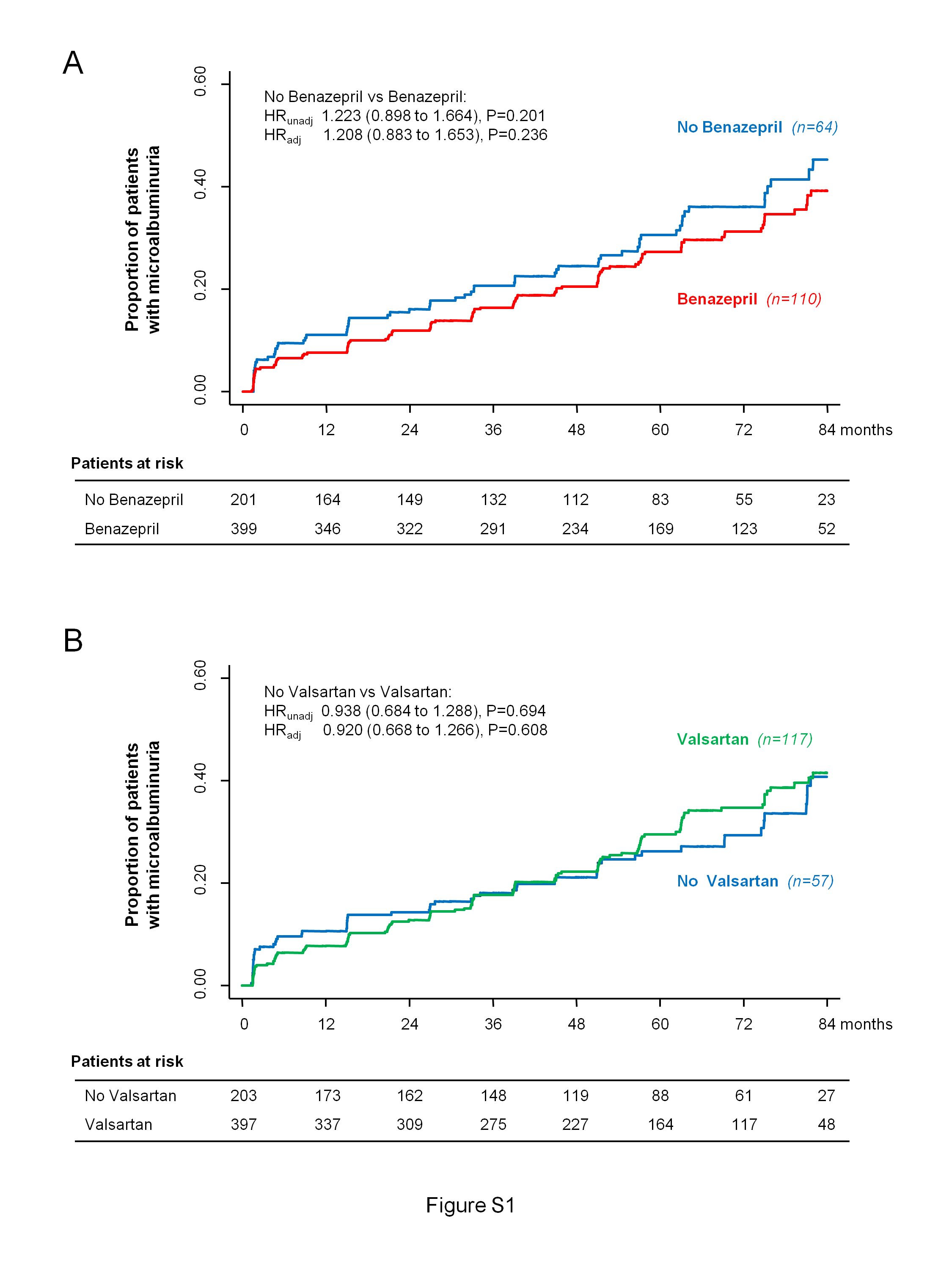

Supplement: S1 Fig — ACE, angiotensin converting enzyme; ARB, angiotensin receptor blocker. (TIF) [file pmed.1003691.s004.tif]

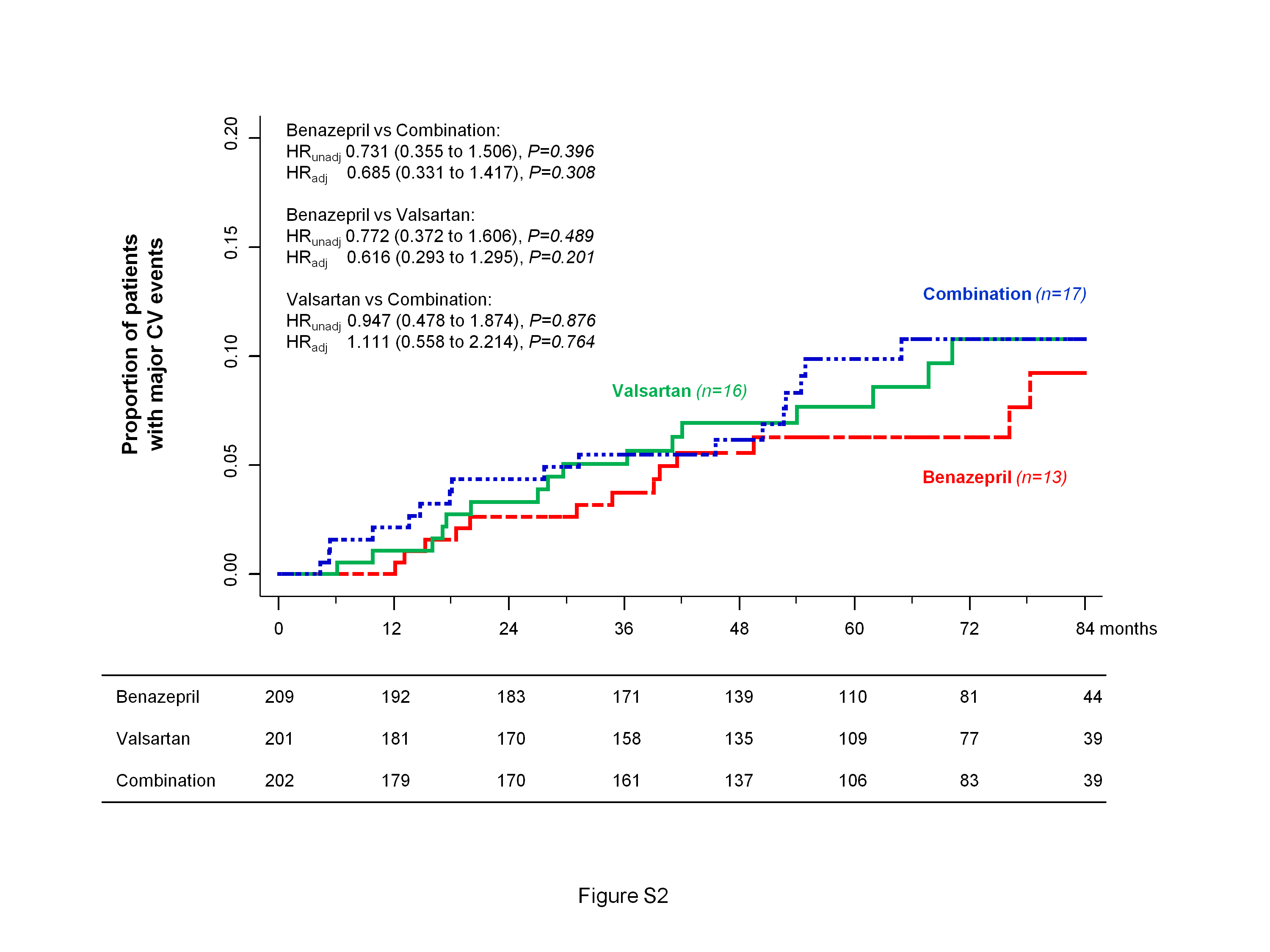

Supplement: S2 Fig — Kaplan–Meier curves show the proportion of patients who reached the exploratory composite cardiovascular endpoint in the benazepril, valsartan, and combination therapy groups during a median follow-up of 66 months. HRs and 95% confidence intervals are crude (unadjusted) and adjusted for center, age, sex, smoking habit, baseline mean BP, and log-transformed UAE. Adj, adjusted; BP, blood pressure; HR, hazard ratio; UAE, urinary albumin excretion; unadj, unadjusted. (TIF) [file pmed.1003691.s005.tif]

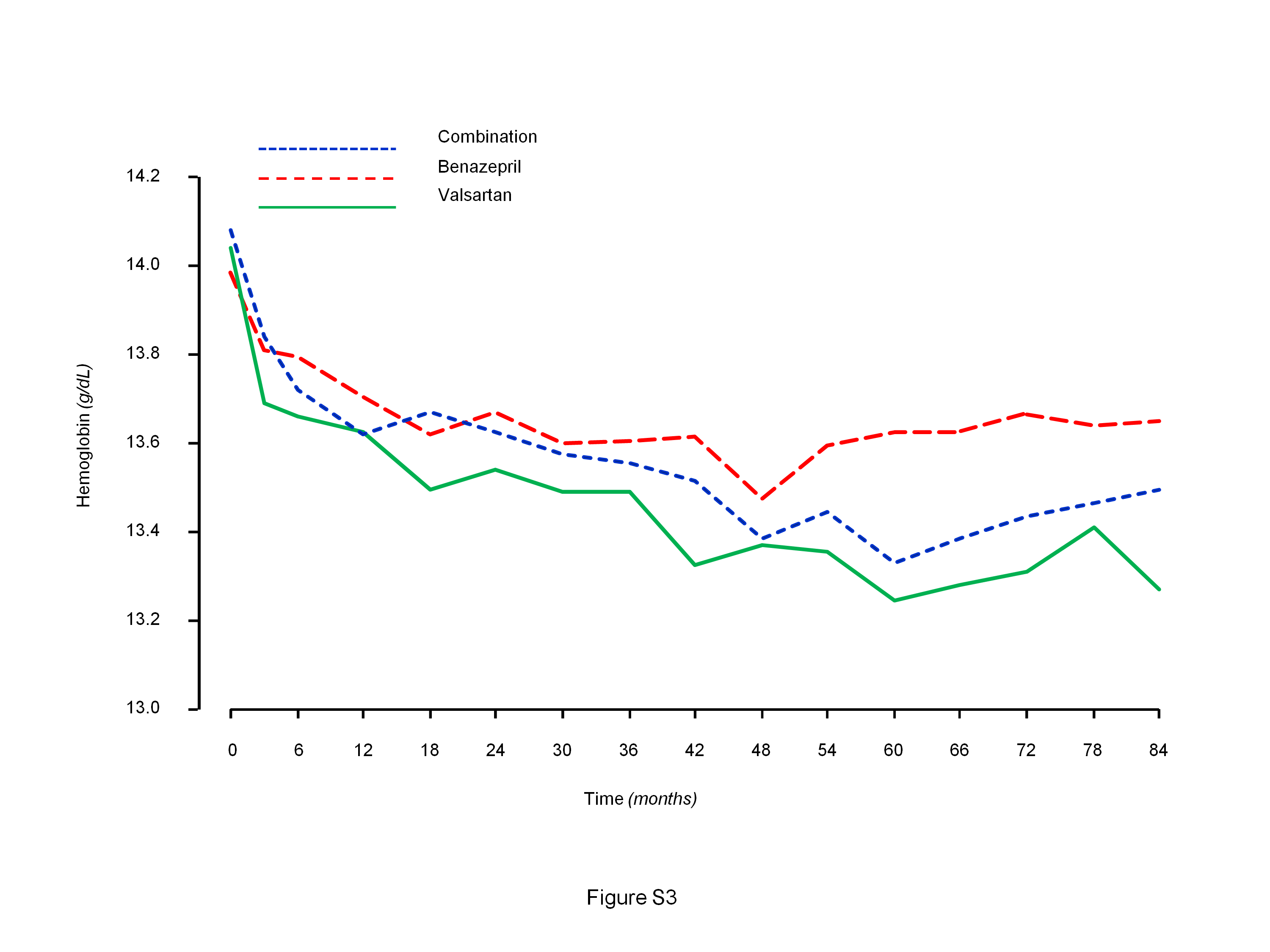

Supplement: S3 Fig — (TIF) [file pmed.1003691.s006.tif]
