# Supplementary material for: Preventing microalbuminuria with benazepril, valsartan, and benazepril–valsartan combination therapy in diabetic patients with high-normal albuminuria: A prospective, randomized, open-label, blinded endpoint (PROBE) study
Source: PLoS Med. 2021 Jul 14;18(7):e1003691. doi: 10.1371/journal.pmed.1003691 (PMC8279302; doi:10.1371/journal.pmed.1003691)
Supplement: S1 Appendix — (PDF) [file pmed.1003691.s007.pdf]

ISTITUTO DI RICERCHE FARMACOLOGICHE MARIO NEGRI

Centro di Ricerche Cliniche per le Malattie Rare "Aldo e Cele Daccò"

Villa Camozzi – 24020 Ranica (BG) – Italy, tel +39.035.45351 - fax +39.035.4535.371
villacamozzi@marionegri.it – http://negribergamo.marionegri.it

A PROSPECTIVE, RANDOMIZED, OPEN LABEL BLINDED END POINT *(PROBE)* TRIAL TO EVALUATE WHETHER, AT COMPARABLE BLOOD PRESSURE CONTROL, COMBINED THERAPY WITH THE ACE INHIBITOR BENAZEPRIL AND THE ANGIOTENSIN II RECEPTOR BLOCKER (ARB) **VA**LSARTAN, **R**EDUCES THE **I**NCIDENCE OF MICROALBUMINURIA MORE **E**FFECTIVELY THAN BENAZEPRIL OR VALSARTAN ALONE IN HYPERTENSIVE PATIENTS WITH **TY**PE 2 DIABETES AND HIGH-NORMAL ALBUMINURIA (VARIETY STUDY)

Running title

Preventing microalbuminuria in type 2 diabetes

Key words: *type 2 diabetes, microalbuminuria, hypertension*

Prepared by

Piero Ruggenenti MD and Giuseppe Remuzzi MD

Mario Negri Institute for Pharmacological Research
and
Unit of Nephrology, Azienda Ospedaliera Ospedali Riuniti,
Bergamo, Italy

Final version

Bergamo, December 15th, 2006

Amended

Bergamo, May 20th, 2015

IRCCS – Decreto Ministeriale 18 gennaio 2013 (Gazzetta Uff. N. 34 del 9/2/2013)
I CONTRIBUTI PER LA RICERCA VERSATI ALL'ISTITUTO SONO FISCALMENTE DEDUCIBILI DAL REDDITO (Gazzetta Uff. N. 179 del 3/8/2011)
FONDAZIONE PER RICERCHE ERETTA IN ENTE MORALE, D.P.R. 361 DEL 5/4/1961 - REGISTRO PERSONE GIURIDICHE PREFETTURA MILANO N.227
CONTO CORRENTE POST. N.58337205 - COD. FISC. E PARTITA IVA 03254210150 - ANAGRAFE NAZIONALE RICERCHE COD.G1690099
RECOGNIZED AS A TAX EXEMPT ORGANIZATION UNDER SECTION 501 (c)(3) OF THE USA INTERNAL REVENUE CODE-TAX I.D. No.: 98-6000957
Istituto con sistema di gestione qualità UNI EN ISO 9001:2008 certificato da Certiquality
(Il dettaglio delle attività oggetto del certificato N. 6121 è disponibile sul sito http://www.marionegri.it/mn/it/sezioni/formazione/index.html)

SEDE LEGALE: Via Giuseppe La Masa, 19 - 20156 Milano MI - Italy

Confidential Statement

This protocol contains strictly confidential information which is not to be communicated or published unless previously authorized by

Mario Negri Institute for Pharmacological Research

Clinical Research Center for Rare Diseases

Synopsis

Background

In people with type 2 diabetes, microalbuminuria is a strong, independent risk factor for diabetic nephropathy and cardiovascular morbidity and mortality. In a recent large scale clinical trial [BErgamo NEphrologic DIabetes Complication Trial (BENEDICT)] ACE inhibitor therapy decreased the risk of microalbuminuria in hypertensive subjects with type 2 diabetes and normoalbuminuria by about 40%. Available data suggest that angiotensin II receptor blockers (ARBs) might have a similar renoprotective effect and that this effect might be increased by combined ACE inhibitor therapy.

Objectives

The study will evaluate the effects, at similar blood pressure control (systolic/diastolic <130/80 mmHg), of dual renin-angiotensin-system (RAS) blockade by benazepril and valsartan combination therapy as compared to single RAS blockade by benazepril or valsartan alone on microalbuminuria and cardiovascular events in high-risk patients with type 2 diabetes and no evidence of renal disease. The relationships between albuminuria and cardiovascular outcomes will also be evaluated.

Methods

This will be a multicenter, Prospective, Randomized, Open label, Blinded End point (PROBE) trial of at least 3-year treatment with halved doses of benazepril (10 mg/day) and valsartan (160 mg/day) given in combination, or full doses of both benazepril (20 mg/day), or valsartan (320 mg/day) given alone in 564 consenting patients >40 year old, with type 2 diabetes (WHO criteria), serum creatinine ≤1.5 mg/dl, high-normal albuminuria (UAE ≥7 and <20 µg/min in at least 2 of 3 overnight urine collections), and no specific contraindications to the study drugs. Primary outcome variable will be microalbuminuria (UAE ≥ 20 µg /min in at least 2 of 3 overnight urine collections in two consecutive visits 2 months apart) and primary comparison will be between the combined benazepril plus valsartan and the benazepril alone groups. The analysis will have an 80% power to detect (a=0.05, two-side test) a 40% difference in the incidence of microalbuminuria.

Expected results

The study is expected to show a more effective prevention of microalbuminuria and cardiovascular events with combined than with single drug ACE inhibitor or ARB therapy. As compared to ACE inhibitor, ARB therapy is expected to have a similar effect on microalbuminuria, but an inferior cardioprotective effect. Applied to clinical practice, the findings should help preventing renal and cardiovascular complications, and related treatment costs, of type 2 diabetes.

BACKGROUND

Nephropathy of type 2 diabetes is the leading cause of end stage renal disease (ESRD) (1). Currently, more than 50% of patients on renal replacement therapy in the US are diabetics. The yearly incidence of diabetics progressing to ESRD and the proportion of ESRD patients with diabetes is progressively increasing due to the ongoing epidemic of type 2 diabetes. According to the World Health Organization (WHO), diabetes affects over 170 million people, and, due to progressive ageing of the population, life-style modifications and increasing burden of obesity, this number is expected to rise to 370 millions by 2030 (2) About one third of those affected will eventually have progressive deterioration of renal function (1) Untreated, these patients progress to ESRD within 10 years or die prematurely because of cardiovascular complications (that are 10-fold more frequent in diabetics with nephropathy than in those without and the general population) (1). Costs for renal replacement therapy of these patients – and for treatment of related complications - are also progressively increasing. Where renal replacement therapy is available only for a small minority of subjects, these patients already die because of uremia.

The first clinical sign of renal dysfunction in diabetic patients is persistent microalbuminuria (3) which develops in 2 to 5 percent of patients per year (4,5) and progresses to overt proteinuria in 20 to 40 percent of cases (6-7). In 10 to 50 percent of patients with proteinuria, chronic kidney disease develops that ultimately requires dialysis or transplantation (8-10). Of great concern, 40 to 50 percent of type 2 diabetic patients with microalbuminuria eventually die of cardiovascular disease (6,11,12). This is three times as a high rate of death from cardiac causes as among patients who have diabetes but have no evidence of renal disease (5). Thus, preventing (or delaying) the development of microalbuminuria is a key treatment goal for renoprotection and, possibly, for cardioprotection.

A small study found that the ACE inhibitor enalapril decreased the incidence of microalbuminuria in patients with type 2 diabetes and normal blood pressure, as compared with placebo (13). This study, however, included only non obese subjects. Most important, the blood pressure was lower in patients on enalapril than in those on placebo throughout most of the study period and the study was underpowered to evaluate whether the effect of enalapril in preventing microalbuminuria was explained by ACE inhibition or, rather, by more effective blood pressure control. More recently, the BErgamo NEphrologic DIabetes Complications Trial (BENEDICT) found that treatment with the ACE-inhibitor trandolapril (either alone or combined to the calcium channel blocker verapamil) significantly reduced the incidence of microalbuminuria in 1204 patients with type 2 diabetes and hypertension, but normal urinary albumin excretion, as compared with placebo (14). The effect of preventing microalbuminuria exceeded expectations based on changes in blood pressure alone and was not enhanced by combined calcium channel blocker therapy.

Large-scale randomized trials evaluating the effects of angiotensin receptor blockers (ARBs) on the incidence of microalbuminuria in type 2 diabetic patients with normal urinary albumin excretion rate are missing. However, studies in patients with type 2 diabetes and micro- (15) or macro- (8,9) albuminuria clearly show that ARBs may shear with ACE inhibitors a similar renoprotective effect in this typology of patients. Indeed, ARBs decreased the incidence of macroalbuminuria in patients with microalbuminuria (15), and slowed the progression to ESRD in those with macroalbuminuria at study entry (8,9). Whether the beneficial effect against the development of microalbuminuria is enhanced when ACE inhibitors and ARBs are given in combination is not established so far. However, finding that combined ACE inhibitor and ARB therapy more effectively than single drug RAS blockade reduced albuminuria (16) or proteinuria (17) in subjects with type 2 diabetes and slowed progression to ESRD in those with non-diabetic chronic nephropathies (18), suggests that the renoprotective effect of combined therapy could be superior to that of single drug blockade of the RAS also in diabetic patients with no evidence of renal disease.

A randomized trial powered to detect a reduced incidence of microalbuminuria with combined ARB and ACE inhibitor therapy as compared to ACE inhibition or ARB alone would require several thousands of patients. However, identifying high risk patients who may benefit the most of nephroprotective therapy would allow to design an adequately powered trial with remarkably less patients. Post hoc analyses of the BENEDICT trial (14) found that high-normal albuminuria at baseline evaluation (defined as a urinary albumin excretion rate between 7 μg/min and the upper limit for study entry: 19 μg/min) was the strongest baseline predictor of subsequent development of microalbuminuria. Indeed, regardless of treatment randomization, 86 of 441 (19.5%) patients with high-normal albuminuria (urinary albumin excretion ≥7μg/min) progressed to the end point as compared to only 15 of 763 (2.0%) with low-normal albuminuria (<7μg/min). Thus, large-part of the excess risk for microalbuminuria observed in people with type 2 diabetes is associated with high-normal albuminuria.

Thus, whether dual more than single drug RAS blockade reduces the incidence of microalbuminuria in patients with type 2 diabetes and high normal albuminuria, and whether ACE inhibitors shear with ARBs a similar beneficial effect in this typology of patients is worth investigating. In addition, this clinical trial might have a clinical relevance for the Italian National Health service (SSN): 1. Effective prevention of microalbuminuria in people with type 2 diabetes is expected to translate into a remarkable reduction in subsequent development of overt nephropathy and progression to ESRD, as well as in the excess cardiovascular morbidity and mortality associated with microalbuminuria. 2. A better definition of the cost/effectiveness profile of different study treatments may help optimizing the allocation of available resources in order to achieve more effective prevention at lower costs and at third identifying subjects at increased risk that may benefit the most of intensified treatment, should translate in a further optimization of the use of available resources at population level. Moreover the present study will allow us to compare the

renoprotective effects of equivalent doses of ACE inhibitors or ARBs. Indeed trials formally comparing the effects of these two drugs in people with type 2 diabetes and overt nephropathy are missing so far. A non-inferiority study in type 2 diabetics with micro- or macro-albuminuria found a more consistent GFR reduction over 5 year follow up in patients on ARB than in those on ACE inhibitor therapy (19). However, since the observed difference was inferior to the difference *a priori* considered as statistically significant, it was concluded that ARB therapy was not inferior to ACE inhibitor therapy in providing long-term renoprotection in persons with type 2 diabetes. On the other hand, on the basis of results of available trials in type 2 diabetic patients with micro- or macroalbuminuria (8,9,15), ARBs are currently claimed as first line therapy for people with diabetes and, despite the higher costs, are progressively replacing ACE inhibitors in clinical practice. However, whether this is effectively the intervention strategy with the highest benefit for the patient and the best cost/effectiveness profile for the National Health Care System, remains elusive.

AIMS

Primary

To evaluate whether, at comparable blood pressure control, dual RAS blockade with combined therapy with halved doses of benazepril (10 mg/day) and valsartan (160 mg/day) reduces the incidence of microalbuminuria more effectively than single drug RAS blockade by full doses of benazepril (20 mg/day) given alone in high-risk patients with type 2 diabetes, hypertension and high normal albuminuria.

Secondary

- To evaluate whether, at comparable blood pressure control, dual RAS blockade with combined therapy with halved doses of benazepril (10 mg/day) and valsartan (160 mg/day) reduces the incidence of microalbuminuria more effectively than valsartan (320 mg/day) given alone in high-risk patients with type 2 diabetes, hypertension and high normal albuminuria.
- To evaluate whether, at comparable blood pressure control, the effects of benazepril and valsartan therapy are similar or whether, alternatively, one of the two treatments offer a superior protective effect against the development of microalbuminuria in the above study population.
- To evaluate the effects of the three study treatments on the incidence of fatal and non-fatal cardiovascular events, regression to low-normal albuminuria (urinary albumin excretion <7µg/min), albuminuria (considered as a continuous variable), serum creatinine, lipid profile, and GFR (in a representative subgroup);
- To assess the relationships, in the study group as a whole and within each treatment group, between renal outcome variables (including microalbuminuria and time-dependent changes in albuminuria) and fatal and non-fatal cardiovascular events, between achieved blood pressure or metabolic control and

renal and/or cardiovascular outcome variables and between achieved albuminuria reduction or residual follow-up albuminuria and renal and/or cardiovascular outcome variables.

PATIENTS

Patients will be identified among the subjects who are referred to the Clinical Research Center for Rare Diseases Aldo & Cele Daccò of the Mario Negri Institute for Pharmacological Research and to the Diabetology Units of the Centers involved in the study. The Coordinating Center will include and randomize 300 patients.

Inclusion criteria

- Males and females >40 years old;
- High-risk subjects with type 2 diabetes (WHO criteria);
- History of diabetes not exceeding 25 years;
- High blood pressure (systolic and/or diastolic blood pressure >135/85 mmHg or concomitant treatment with blood pressure lowering medications);
- Serum creatinine concentration <1.5 mg/dl;
- Overnight urinary albumin excretion (in at least 2 of 3 consecutive overnight urine collections) ≥7 and <20 µg/min;
- Legal capacity;
- Written informed consent.

Exclusion criteria

- Uncontrolled diabetes (glycated hemoglobin >11%);
- Specific contraindications or history of hypersensitivity to the study drugs;
- Serum potassium ≥ 5.5 mEq/L despite diuretic therapy, and optimized metabolic and acid/base control;
- Bilateral renal artery stenosis;
- Previous history of allergy or intolerance, or evidence of immunologically-mediated renal disease, systemic diseases, cancer;
- Drug or alcohol abuse;
- Any chronic clinical conditions that may affect completion of the trial or confound data interpretation;
- Pregnancy or lactating;
- Women of childbearing potential without following a scientifically accepted form of contraception;
- Legal incapacity and/or other circumstances rendering the patient unable to understand the nature, scope and possible consequence of the trial;
- Evidence of an uncooperative attitude;
- Any evidence that patient will not be able to complete the trial follow-up.

RANDOMIZATION

Randomization will be centralized at the Laboratory of Biostatistics of the Clinical Research Center for Rare Diseases "Aldo e Cele Daccò" Villa Camozzi, Ranica, Bergamo of the Mario Negri Institute for Pharmacological Research under the responsibility of an independent investigator. Randomization will be stratified in blocks by center. The block size will randomly vary in order to increase the unpredictability of the sequence.

STUDY DRUGS

Study drugs (benazepril: 5 - 10 mg tablets; valsartan: 80 - 160 mg capsules) will be manufactured by Meda Pharma SpA (Viale Brenta 18, Milano) and Novartis Farma SpA (Largo Umberto Boccioni 1, Origgio, Varese), respectively.

Boxes will be labeled with "tear-off" labels. When administering the study medication, the Investigator will peel off the respective part of the label from the each box and will fix these to the provided space in the appropriate form.

All medication supplies must be stored separately from normal hospital/practice stocks. The study medication can remain below 30°C.

The Investigator or the hospital pharmacist will confirm receipt and disposition of the study drug in writing, including all following supplies. Unused drug has to be returned to the MNI. The Investigator will administer the study drug only to the patients included in the trial by following the procedures set out in the study protocol. It is prohibited to use the trial drug for any other purpose.

STUDY DESIGN AND TREATMENT

This will be a Prospective, Randomized, Open label, Blinded End point (PROBE) trial (20) of 3-year treatment with the ACE inhibitor benazepril and the ARB valsartan given alone or in combination. After one month wash-out from previous RAS (ARB or ACE) inhibitor therapy, patients satisfying the inclusion/exclusion criteria will be randomly given equivalent doses (half the standard doses recommended by the manufacturer for blood pressure control) of benazepril (10 mg/day) or valsartan (160 mg/day) or one fourth of the standard doses of both agents in combination (benazepril 5 mg/day and valsartan 80 mg/day). Blood pressure, serum creatinine, serum potassium, blood glucose and venous pH will be monitored within 7-10 days after randomization. If well tolerated, treatment will be up-titrated to full dose of benazepril (20 mg/day) or valsartan (320 mg/day) or one half of the standard doses of both agents in combination (benazepril 10 mg/day and valsartan 160 mg/day). Blood pressure, serum creatinine, serum potassium, blood glucose and venous pH will be monitored within 7-10 days after up-titration of the study treatments. Target blood pressure will be 130/80 mmHg in all treatment groups. Additional treatment with thiazide or loop diuretics (first step),

beta and/or alpha blockers or clonidine (second step) and vasodilators such as dihydropyridine calcium channel blockers or minoxidil (third step) will be allowed to achieve and maintain the target blood pressure. Back titration of concomitant treatments and, secondarily, of the study drugs will be allowed to avoid symptomatic hypotension or severe hyperkalemia (serum potassium ≥ 5.5 mEq/L despite diuretic therapy, and optimized metabolic and acid/base control). If the above clinical or laboratory abnormalities will not recover despite back-titration, the study drugs will be withdrawn. The patients will be monitored until full recovery of the event and, however, for at least two weeks and than withdrawn from the study. The general aim will be to maintain the target blood pressure with the highest dose of the study drugs and the lowest doses of the concomitant medications. Potassium sparing diuretics, aldosterone antagonists and RAS inhibitors different from the study drugs will not be allowed. Diet, antidiabetic and statin therapy will also target at optimized metabolic (HbA1C<7%) and lipid (total cholesterol <200 mg/dL) control in all patients.

Glycated hemoglobin, lipids, the glomerular filtration rate (GFR, in a subgroup), and routine clinical and laboratory parameters will be evaluated at randomization, 3 and 6 months after randomization and every 6 months up to study end. The measurements of albuminuria (by nephelometry) and GFR [by iohexol plasma clearance (21)] will be centralized at the laboratory of our Clinical Research Center. This laboratory already served for centralized measurements of GFR [by a technique that has been set up by the head of the laboratory (21)] in previous multicenter, randomized trials in non-diabetic renal disease (22-24) and for all routine laboratory tests including albuminuria, lipids, hematochemistry, blood cell counts in a previous large scale, multicenter, randomized trial in patients with type 2 diabetes (14), as well as in other ongoing clinical trials in a similar typology of patients. All the investigators involved in laboratory measurements will be blinded to the treatment assignments of the subjects.

All clinical and laboratory parameters as well as any serious and non serious adverse event will be recorded throughout the study for safety analyses.

MAIN OUTCOMES

Variables

Primary

Development of persistent microalbuminuria (i.e. urinary albumin excretion rate >20 µg/min in at least 2 of 3 consecutive overnight urine collections confirmed in two consecutive visits). Whenever a patient will be found to have 2 of 3 collections in the microalbuminuric range, he will be asked to submit 3 additional consecutive collections within 2 months. If urinary albumin excretion rate is still >20 µg/min in at least 2 of 3 samples, he will be considered to be microalbuminuric and to have reached the end point.

Secondary

- Regression to low-normal albuminuria (i.e. urinary albumin excretion rate <7 µg/min in at least 2 of 3 consecutive overnight urine collections confirmed in two consecutive visits);
- Albuminuria (considered as a continuous variable);
- Serum creatinine (versus baseline);
- Incidence of fatal and non-fatal cardiovascular events (stroke, acute myocardial infarction, sudden death)

Safety variables

They will include serious and non-serious adverse events and any clinical or laboratory abnormality (such as acute hypotension, cough, acute renal function deterioration or hyperkalemia) possibly related to the study drugs.

SAMPLE SIZE

Primary efficacy comparison will be between combined ACE inhibitor plus ARB therapy and ACE inhibitor alone. On the basis of the outcome data of the BENEDICT study (14) and the first interim analysis 6% of patients with high normal albuminuria are expected to progress to microalbuminuria every year. It is predicted that at the time the last randomized patient will have completed the planned 3-year treatment period (study end), the median follow-up of the whole study group will approximate 4.5 years. Thus, assuming a 6% yearly incidence of microalbuminuria and a 4.5-year follow-up, 27% of patients in the ACE inhibitor group are predicted to develop microalbuminuria throughout the study period. Similar figures are expected for patients in the ARB group. On the basis of preliminary evidence in type 2 diabetes (10) this figure is, conservatively, predicted to decrease by 45% (from 27% to 14.85%) by combined therapy. To give the trial an 80% power to detect by a two-side test with a type 1 error of 5% the expected reduction in microalbuminuria events, 179 patients per group should complete the study. Thus, to account for a 5% drop-out rate, 188 patients per group should be included for a total of 564 patients. Assuming a similar incidence of events in the ACE inhibitor and ARB groups, this sample size should provide adequate power to detect a difference, if any, also between the combined and the ARB inhibitor treatment group. Comparative analyses will consider also the ACE inhibitor and the ARB arms. No sample size, however, is calculated for this comparison.

STATISTICAL ANALYSIS

The main treatment group comparison is between dual RAS blockade with combined therapy with halved doses of benazepril (10 mg/day) and valsartan (160 mg/day) vs full doses of benazepril (20 mg/day), and the 5% level of significance in a two-sided test will be used for this comparison. Secondary comparisons will be a. combined therapy vs full doses of valsartan (320 mg/day) and b. full doses of benazepril (20 mg/day) vs full

doses of valsartan (320 mg/day). These descriptive comparisons will be according to a two-sided test and conducted at the 2.5% level of significance (two-sided test) to take account of multiple testing.

Statistical analysis will be performed according to the intention-to-treat principle. Statisticians and investigators involved in statistical analyses will be blinded to the treatment assignments of the subjects. Primary end point is time to progression to microalbuminuria. At variance with end points such as 'death' or 'dialysis', the microalbuminuric state does not allow a clear cut recognition of the exact occurrence date, but, rather, two dates can be identified: the last time the patient is found to be normoalbuminuric and the first time the patient is detected as microalbuminuric. This kind of end point gives rise to interval censored data. The interval censoring method will take into account that the 'true' date of microalbuminuria development lies within this interval. The primary analysis will be performed by means of the accelerated failure time model, implemented in SAS PROC LIFEREG, which allows direct incorporation of interval censored data, and by use of proportional hazards model, implemented in SAS PROC PHREG. The following baseline covariates will be included in the model: site, age, gender, smoking habits (never vs. current and former), a measure of blood pressure and log transformed urinary albumin excretion. Kaplan-Meier curves will also be plotted, using the midpoint of the interval as event time. One interim analysis will be undertaken on the intention-to-treat population, when about 50% of total information is expected to be collected. The method of O'Brien and Fleming will be used to determine for statistical significance at the interim evaluation, that would constitute grounds to recommend trial termination. This threshold will be a P value of 0.0054 (nominal significance level) or less in order to 'preserve' an overall 5% level of significance. A further evaluation will take place at the end of the enrolment period for the purposes of possible adaptation of the sample size or observation period in order to achieve adequate power. Other details regarding the statistical approaches will be supplemented by a detailed plan which will be prepared after release of the protocol but before the start of the clinical part of the study. Should new evidence come to light as a result of running other trials before this trial is analyzed or should new methods of analysis become available in the statistical literature, or should the trial be affected by unforeseen circumstances, it may be necessary to modify the analysis plan.

CENTERS

The study will be coordinated by the Clinical Research Center for Rare Diseases of the Mario Negri Institute for Pharmacological Research, Ranica, Bergamo and will be conducted with the cooperation of the Units of Diabetology and of Nephrology of the Azienda Ospedaliera "Ospedali Riuniti" of Bergamo, the Department of General Medicine of the "Ospedale San Raffaele" of Milan, the Department of Clinical Research and Experimental Medicine of the University of Padua, the Unit of Diabetology of the Treviglio Hospital and the other Units of Diabetology of the province of Bergamo included in the BENEDICT network[18]. Data handling will be performed by Mario Negri Institute (MNI) Coordinating Center.

The study will be overseen by Steering, Safety and End-Point Committees. The Steering Committee will be chaired by the principal investigator and will include five voting members (the chair, one nephrologist, one diabetologist, one statistician and one research nurse). Three members of the Ethics Committee (see description below) will act as Safety Committee, which will include two medical doctors and one statistician, not otherwise involved in the conduct of the study. They will periodically review, in an unblinded fashion, the serious adverse events and the report of the interim analysis on efficacy data. For the above evaluation the Laboratory of Biostatistics of the Mario Negri Institute will provide the Safety Committee with the randomization list of the study. The End Point Committee will include three independent external reviewers (one nephrologist, one diabetologist and one cardiologist) who are not members of the Steering and Safety Committees and who will blindly and individually review all primary and secondary end points. Any discrepancies will be adjudicated after discussion during regular meetings. In order to ensure blinding of the End Point Committee, all end point reports accompanied by a patient's documentation (e.g narratives or copies of hospital records) will be previously sent to an external data center for blinding.

DATA HANDLING AND RECORD KEEPING

Case report forms completion

CRFs will be supplied by the MNI Coordinating Center. Demographic, efficacy and safety data will be collected for the purpose of the study, to be documented by the investigator or his/her designated on the individual case report form (CRF). This also applies to the data for patients who, after having consented to participate, underwent baseline examinations but were not further randomized. The investigator must be provided with a CRF copy. To ensure CRF legibility, CRFs should be filled out in block capitals with a black/blue ball point pen. Any CRF corrections must be carried out by the investigator. The correction has to be dated and initialled. A reasonable explanation must be given by the investigator for all missing data. All medical records upon which the CRFs are based must be kept for at least 15 years after completion of the study. The investigator will be responsible for the accuracy of the data entered in the CRFs. All entries must be written in ENGLISH in black ink. Source documents should be available to support all the data recorded in the CRFs.

Location of source data, including those for which the CRF might be accepted as being the sole source document, will be specified and listed at the Center initiation visit.

The CRF must be available for review/collection to designated MNI Coordinating Center Representatives at each scheduled monitoring visit.

STUDY MONITORING

The study will be monitored by the staff of the Mario Negri Institute Drug Monitoring Unit. The Investigator agrees to allow monitoring visits on site (clinical area and laboratory) at regular times prior, during and after the completion of the study. These visits are designed to implement Good Clinical Practice requirements.

The study will be monitored by means of on-site visits, telephone calls and regular inspection of the case report forms with sufficient frequency to inspect the following: patient enrolment, compliance with the protocol procedures, the completeness and exactness of data entered on the case report forms, verification against original source documents, and occurrence of adverse events. Data verification is required and will be done by direct comparison with source documents in case of patient's respective consent or by cross-checking with source documents always giving due consideration to data protection and medical confidentiality.

The Investigator will ensure that adequate time will be available for the Clinical Monitor to review case report forms, study source and raw data, and to assure the accuracy and completeness of the recorded data. The Clinical Monitor will compare the data in the case report forms with source document(s).

During the visits, the Clinical Monitor will ensure that there is adherence to the protocol, and that patients included in the study meet the inclusion criteria and that patients and/or caregivers have given their written informed consent. The Clinical Monitor will collect completed and signed case report forms as they become available.

DATA MANAGEMENT

In order to ensure that the data base accurately reflects data of the case report forms, a double entry procedure will be used.

The data entry will be made by the staff of the Mario Negri Institute Drug Monitoring Unit. Data will be entered by different staff members in the same data base. Inconsistencies derived from a computer-assisted comparison of these two data entries will be carefully clarified. In addition, a systematic and extensive electronic validation of the quality of the data base will take place.

Pre- and concomitant medication, concurrent diseases as well as adverse events will be coded according to standardized dictionaries. Once all data discrepancies will be corrected, the database will be locked and data converted into SAS-transport files. Due to the PROBE design of the study[20], only those assessing the outcomes will be blinded to group assignment.

ESSENTIAL DOCUMENT RETENTION

The investigator will retain copies of all the essential documents as required by the applicable regulatory requirements. The investigator should take measures to prevent accidental or premature destruction of these documents.

The essential documents include at least: the signed protocol, copies of the completed CRFs, signed patient informed consent documents from all patients who consented, Hospital records, and other source documents, IEC approval and all related correspondence, including approved documents, and all other documentation included in the investigator site file and pharmacy/dispensing file.

ADVERSE EVENT DEFINITION

Adverse Event

Any untoward medical occurrence in a patient or clinical investigation subject administered a pharmaceutical product and which does not necessarily have to have a causal relationship with this treatment.

An adverse event (AE) can therefore be any unfavourable and unintended sign (including an abnormal laboratory finding, for example), symptom, or disease temporally associated with the use of a medicinal product, whether or not considered related to the medicinal product.

Adverse Drug Reaction

An adverse drug reaction (ADR) is a noxious and unintended response to a medicinal product related to any doses. The phrase “response to a medicinal product” means that a causal relationship between a medicinal product and an adverse event is at least a reasonable possibility, i.e. the relationship cannot be ruled out.

Serious Adverse Event

A serious adverse event is any untoward medical occurrence that at any dose:

- results in death,
- is immediately life-threatening,

  NOTE: the term "life-threatening" in the definition of "serious" refers to an event in which the patient was at risk of death at the time of the event; it does not refer to an event which hypothetically might have caused death if it were more severe.
- requires inpatient hospitalisation or prolongation of existing hospitalisation,

  a) elective surgery or cases where the decision to hospitalize the patient was made before the signed consent or attendance at hospital without an overnight stay, is not hospitalisation

  b) event which would have normally required hospitalisation, but the patient was not hospitalised (for whatever reason) is hospitalisation

  c) if the patient was already in the hospital when the event occurred, and the event required inpatient treatment (i.e. the patient would have been hospitalized unless she/he already was) but did not cause the prolongation of the hospitalization, the event must be reported as serious for hospitalization;
- results in persistent or significant disability/incapacity, or
- is a congenital malformation/birth defect.

- other: it is a category that covers events that are considered as serious, when based upon appropriate medical judgment that they may jeopardise the patient/subject, and may require medical or surgical intervention to prevent one of the other seriousness criteria from occurring;
  - patient requiring treatment in order to prevent serious outcome: if a patient required treatment but not hospitalisation in order to prevent permanent and/or severe disablement or fatal outcome, then the event is reported per serious standard. Treatment may involve a physician's visit or presentation to the emergency room.
  - cancer: if cancer is the indication for treatment , only cancers of new histology and cases where there is clear evidence of exacerbation of an existing cancer qualify as a serious event. Every new occurrence of cancer must be reported as a serious event regardless of the duration between discontinuation of the drug and the occurrence of the cancer.
  - abuse or dependency: it qualifies as serious for other medical reasons

An event which doesn't meet these definitions is considered to be "Not Serious".

COLLECTION/REGISTRATION OF INFORMATION

Adverse events will be monitored at each visit and by telephone reports from responsible caregivers as needed between outpatient visits.

Any adverse event reported spontaneously or upon questioning will be evaluated by the Investigator and recorded on the relevant page in the case report form.

However, if the event includes a group of signs and symptoms, which, when considered together, are known or presumed to characterize a syndrome, only one form must be filled in. In this case, all events will be fully described, but any additional information such as intensity, outcome, causality, etc., will refer to the syndrome only. For each adverse event the Investigator is required to evaluate the nature of the event, the date and time of its onset (if known), the date and time the adverse event stopped (if known), the duration of the event and its maximum intensity. The Investigator will record any remedial action taken and the final outcome of the adverse event. All modern facilities for resuscitation must be immediately available in case of emergency. The Investigator is also required to assess the causal relationship between the event and the study treatment.

All the patients experiencing adverse events must be monitored until symptoms and any abnormal laboratory values have returned to baseline, or until there is a satisfactory explanation for the changes observed, or until death, in which case a full pathologist's report should be supplied, if possible. All findings must be recorded on the "adverse event" page in the case report forms and in the patients' medical records.

In case of serious adverse events, the following reporting procedure has to be followed: for all serious adverse events which occur during the study whether considered to be associated with the trial drug or not, the Investigator must send the initial SAE report immediately (within 24 hours from the knowledge of the event),

by fax to Mario Negri Institute; the investigator has to document the event also on an “adverse event” page in the case report forms.

If necessary, one or more follow-up reports including all additional information obtained on the serious adverse event must be prepared by the Investigator.

The contact addresses for reporting of serious adverse events is:

Nadia Rubis, *Clinical Monitor*

Drug Monitoring Unit

Istituto di Ricerche Farmacologiche Mario Negri

Centro di Ricerche Cliniche per le Malattie Rare

Aldo e Cele Daccò

Via G. B. Camozzi, 3

I-24020 Ranica (Bergamo)

Italy

**SAEs Fax:**	**+39 035 4535371**
Tel:	+39 035 45351
e-mail:	farmacovigilanza.vc@marionegri.it

LEGAL AND ETHICAL ASPECTS

Health Authorities and Ethics Committee

The study protocol will be in accordance with the declaration of Helsinki and will be approved by the institutional review board at each center and by the safety committee of the study group. The study protocol and all the other appropriate documents will be submitted to the Regulatory Authorities in accordance with local legal requirements. The investigational sites will not commence study procedures until has received the appropriate written approval.

All protocol amendments, treatment-related serious adverse events and new versions of the Investigator’s Brochure must be submitted to the Principal Investigators and to the Ethics Committee.

Written Informed Consent

Good Clinical Practice guidelines require that a written informed consent be obtained from each patient.

Doctor must inform patient and caregiver of the aims of the study and how it will be organized, the type of study treatments, the anticipated benefits which can be expected from the study, any potential hazards of the study and discomfort it may entail, alternative treatments, the freedom to ask for further information at any time, the patient’s right to withdraw from the trial at any time without giving reasons and without jeopardizing the further course of the treatment, the existence of patient’s insurance cover and obligations following from

this cover. This information should be given in both oral and written form. The patient and the caregiver should have sufficient time to decide whether or not to take part in the study.

Insurance policy

The study is covered by an insurance policy, according to the laws in force, in the event of a patient suffering any significant deterioration in health or well-being, which is proven as being as a direct result of study participation.

REFERENCES

1. Remuzzi G, Schieppati A, Ruggenenti P. Nephropathy in patients with type 2 diabetes. New Engl J Med 2002;346:1145-1151.
2. World Health Organization. The Diabetes Program 2004. (Accessed September 21, 2004, at http//www.who.int/diabetes/en/.).
3. Ruggenenti P, Remuzzi G. The diagnosis of renal involvement in non-insulin-dependent diabetes mellitus. Curr Opin Nephrol Hypertens 1997;6:141-145.
4. Gall MA, Hougaard P, Borch-Johnsen K, Parving HH. Risk factors for development of incipient nephropathy in patients with non-insulin dependent diabetes mellitus: prospective, observational study. Br Med J 1997;314:783-788.
5. Adler AI, Stevens RJ, Manley SE, Bilous RW, Cull CA, Holman RR on behalf of the UKPDS Group. Development and progression of nephropathy in type 2 diabetes: the United Kingdom Prospective Diabetes Study (UKPDS 64). Kidney Int 2003;63:225-232
6. Mogensen CE. Microalbuminuria predicts clinical proteinuria and early mortality in maturity onset diabetes. N Engl J Med 1984;310:356-360
7. Nelson RG, Knowler WC, Pettit DJ, Saad MF, Charles MA, Bennett PH. Assessing risk of overt nephropathy in diabetic patients from albumin excretion in untimed urine specimens. Arch Intern Med 1991,151:1761-1765.
8. Brenner BM, Cooper ME, de Zeeuw D, et al. Effects of losartan on renal and cardiovascular outcomes in patients with type 2 diabetes and nephropathy. New Engl J Med 2001;345:861-869.
9. Lewis EJ, Hunsicker LG, Clarke WR, et al. Renoprotective effect of the angiotensin receptor antagonist irbesartan in patients with nephropathy due to type 2 diabetes. New Engl J Med 2001;345:851-860.
10. Nelson RG, Newman JM, Knowler WC et al. Incidence of end stage renal disease in type 2 (non insulin dependent) diabetes mellitus in Pima Indians. Diabetologia 1988, 31:730-736.
11. Eurich DT, Majumdar SR, Tsuyuki Rt, Johnson JA. Reduced mortality associated with the use of ACE inhibitors in patients with diabetes. Diabetes Care, 27:1330-1334.
12. Parving HH, Mauer M, Ritz E. Diabetic nephropathy. In: Brenner BM, ed Brenner & Rector's The kidney.7th ed. Vol.2Philadelphia:Saunders 2004:1777-1818.
13. Ravid M, Brosh D, Levi Z, Bar-Dayan Y, Ravid D, Rachmani R. Use of enalapril to attenuate decline in renal function in normotensive, normoalbuminuric patients with type 2 diabetes mellitus. Ann Intern Med 1998;128:982-988.
14. Ruggenenti P, Fassi A, Parvanova A et al. Preventing microalbuminuria in type 2 diabetes. N Engl J Med 351:1941-1951, 2004

15. Parving HH, Lehnert H, Brochner-Mortensen J, Gomis R, Andersen S, Arner P, for the Irbesartan in Patients with Type 2 Diabetes and Microalbuminuria Group. The effect of irbesartan on the development of diabetic nephropathy in patients with type 2 diabetes. N Engl J Med 2001;345:870-8.
16. Mogensen CE, Neldam S, Tikkanen I, Oren S, Viskoper R, Watts RW, Cooper ME for the CALM study group. Randomised controlled trial of dual blockade of renin-angiotensin system in patients with hypertension, microalbuminuria, and non-insulin dependent diabetes: the candesartan and lisinopril microalbuminuria (CALM) study. BMJ 2000, 321: 1440-1444
17. Parvanova A, Pisoni R, Dimitrov BD, Iliev I, Perna A, Ruggenenti P, Stucchi N, Lutz J, Remuzzi G. Relative renoprotective effect of ACE inhibitors (ACEi), angiotensin II antagonists (ATA), ACEi and ATA combination and dihydropyridine calcium channel blockers (dCCBs) in overt nephropathy of type 2 diabetes. J Am Soc Nephrol 2001; 12:153A
18. Nakao N, Yoshimura A, Morita H, Takada M, Kayano T and Ideura T. Combination treatment of angiotensin-II receptor blocker and angiotensin-converting-enzyme inhibitor in non-diabetic renal disease (COOPERATE): a randomised trial. Lancet 2003, 361:117-124
19. Barnett AH, Bain SC, Bouter P et al: Angiotensin-receptor blockade versus converting-enzyme inhibition in type 2 diabetes and nephropathy. N Engl J Med 351:1952-1961, 2004.
20. Hansson L, Hedner T, Dahlof B. Prospective randomized open blinded end-point (PROBE) study: a novel design for intervention trials. Blood Pressure 1:113-119, 1992
21. Gaspari F, Perico N, Ruggenenti P, Mosconi L, Amuchastegui CS, Guerini E, Daina E, Remuzzi G: Plasma clearance of nonradioactive iohexol as a measure of glomerular filtration rate. *J Am Soc Nephrol* 6:257-263, 1995
22. The GISEN Group (Gruppo Italiano di Studi Epidemiologici in Nefrologia). Randomized, placebo-controlled trial of effect of ramipril on decline in glomerular filtration rate and risk of terminal renal failure in proteinuric, non-diabetic nephropathy. Lancet 1997, 349: 1857-1863.
23. Ruggenenti P, Perna A, Gherardi G, Gaspari F, Benini R, Remuzzi G, on behalf of Gruppo Italiano di Studi Epidemiologici in Nefrologia (GISEN). Renal function and requirement for dialysis in chronic nephropathy patients on long-term ramipril: REIN follow-up trial. *Lancet* 352:1252-1256, 1998
24. Ruggenenti P, Perna A, Gherardi G, Garini G, Zoccali C, Salvadori M, Scolari F, Schena FP, Remuzzi G. Renoprotective properties of ACE-inhibition in non-diabetic nephropathies with non-nephrotic proteinuria. The Lancet 354:359-364, 1999.
25. Remuzzi G, Lesti M, Gotti E et al. Mycophenolate mofetil versus azathioprine for prevention of acute rejection in renal transplantation (MYSS): a randomised trial. Lancet 364:503-512, 2004.

Appendix 1

		WASH-OUT PHASE				TREATMENT PHASE							
**Visit number**		**1**	**2**	**3**		**4**	**5**	**6**	**7**	**8**	**9**[1]	...	**FV**
Time[2]		-1 m	-20 d	0		10 d	20 d	3 m	6 m	12 m	18 m	...	36 m
BASELINE DATA	x				RANDOMIZTION								
COMPLETE PHYSICAL EXAMINATION		x	x	x		x	x	x	x	x	x		x
BLOOD PRESSURE – PULSE RATE – WEIGHT		x	x	x		x	x	x	x	x	x		x
ECG AT REST		x		x				x	x	x	x		x
COMPLETE BLOOD LABORATORY EXAMINATIONS[3]		x		x				x	x	x	x		x
SELECTED BLOOD LABORATORY EXAMINATIONS[4]			x			x	x						
MORNING URINE SAMPLE[5]		x		x				x	x	x	x		x
3 CONSECUTIVE OVERNIGHT URINE COLLECTIONS[6]		x		x				x	x	x	x		x
IOHEXOL PLASMA CLEARANCE[7]				x				x	x	x	x		x
WASH-OUT OF PREVIOUS ACEI AND/OR ARBS DRUGS		x											
INCLUSION/EXCLUSION CRITERIA				x									
RANDOMIZATION				x									
STUDY DRUG SUPPLY				x		x		x	x	x	x		x
STUDY DRUG UP-TITRATION						x	(x)	(x)	(x)	(x)	(x)		
STUDY DRUG COMPLIANCE						x	x	x	x	x	x		x
STUDY DRUG TEMPORARY WITHDRAWALS						(x)	(x)	(x)	(x)	(x)	(x)		
STUDY DRUG PERMANENTLY DISCONTINUATION						(x)	(x)	(x)	(x)	(x)	(x)		x
CONCOMITANT MEDICATION/NON-DRUG THERAPY		x	x	x		x	x	x	x	x	x		x
CONCOMITANT DISEASES		x	x	x									
ADVERSE EVENTS						x	x	x	x	x	x		x
TRIAL END SUMMARY						(x)	(x)	(x)	(x)	(x)	(x)		x
EXTRA VISITS			x	x		x	x	x	x	x	x		x

1 The same scheduled visit will be repeated every six months.
2 **Legenda**: "d" means days, "m" means months.
3 **Complete blood laboratory examinations**: creatinine, urea, uric acid, sodium, potassium, GOT/ALT, GPT/AST, gammaGT, alkaline phosphatase, total bilirubin, CPK, glucose, HbA1c, total cholesterol, LDL-cholesterol, HDL-cholesterol, triglycerides, apolipoprotein A, apolipoprotein B, erithrocytes, hematocrit, hemoglobin, leukocytes, platelets, venous pH, PCR.
4 **Selected blood laboratory examinations**: creatinine, urea, sodium, potassium, glucose, venous pH.
5 **Morning urine sample**: Multistix 10SG.
6 **Consecutive overnight urine collections**: urinary albumin excretion rate (UAE) and urinary albumin excretion (UCE).
7 **Iohexol plasma clearance**: glomerular filtration rate (GFR), in a subgroup only.
