# Supplementary material for: Preventing microalbuminuria with benazepril, valsartan, and benazepril–valsartan combination therapy in diabetic patients with high-normal albuminuria: A prospective, randomized, open-label, blinded endpoint (PROBE) study
Source: PLoS Med. 2021 Jul 14;18(7):e1003691. doi: 10.1371/journal.pmed.1003691 (PMC8279302; doi:10.1371/journal.pmed.1003691)
Supplement: S2 Appendix — (DOCX) [file pmed.1003691.s008.docx]

**VARIETY Study Organization *(number of included patients in brackets)***

Coordinating Centre: Mario Negri Institute for Pharmacological Research IRCCS, Clinical Research Centre for Rare Diseases Aldo e Cele Daccò, Villa Camozzi, Ranica (Bergamo); Chief Investigator: Piero Ruggenenti (Bergamo); Trial coordinator: Giuseppe Remuzzi (Bergamo).

Centers including patients: U.O.C. Malattie Endocrine e Centro Regionale per il Diabete Mellito, ASST Bergamo Ovest, Treviglio and Romano di Lombardia (Antonio Carlo Bossi, Aneliya Ilieva Parvanova, Ilian Petrov Iliev, Svitlana Yakymchuk, n = 167+101); Poliambulatorio extra‐ospedaliero, ASST Bergamo Ovest, Brembate di Sopra (Antonio Belviso, Matias Trillini, Veruska Lecchi n = 99); Unità Malattie Endocrine ‐ Diabetologia, ASST Papa Giovanni XXIII, Bergamo (Roberto Trevisan, Elena Mondo, Maria Carolina Aparicio, Sergio Brescianini n = 98); Clinical Research Center for Rare Disease Aldo e Cele Daccò, Ranica (Norberto Perico, Stefano Rota, Silvia Prandini, Monica Cortinovis, Giulia Gherardi, Daniela Cugini n = 86); Nefrologia e Dialisi, Casa Sollievo della Sofferenza, San Giovanni Rotondo (Salvatore De Cosmo, Anna Rauseo n = 32); Unità Operativa di Diabetologia, Ospedale San Giovanni di Dio, Olbia (Giancarlo Tonolo, Dominika Anna Iwaniszyn, Simonetta Caria n = 28); Unità Operativa di Nefrologia, Ospedale San Raffaele, Milano, IRCCS Ospedale San Raffaele (Paolo Manunta, Gianpaolo Zerbini n = 1); ASST Bergamo Est, Seriate (Ruggero Mangili, Manuela Abbate, n = 58 patients screened at this Centre who were then followed at Aldo e Cele Daccò Center).

Activities of the Clinical Research Center: Monitoring, Drug Distribution and Pharmacovigilance (Nadia Rubis, Wally Calini, Olimpia Diadei, Alessandro Villa, Davide Villa); Database and Data Validation (Davide Martinetti, Sergio Carminati); Randomization (Giovanni Antonio Giuliano); Data Analysis (Annalisa Perna, Francesco Peraro, Tobia Peracchi); Centralized Laboratory Measurements (Flavio Gaspari, Fabiola Carrara, Silvia Ferrari, Nadia Stucchi, Antonio Nicola Cannata); Regulatory Affairs (Paola Boccardo, Sara Peracchi).
