# Supplementary material for: Preventing microalbuminuria with benazepril, valsartan, and benazepril–valsartan combination therapy in diabetic patients with high-normal albuminuria: A prospective, randomized, open-label, blinded endpoint (PROBE) study
Source: PLoS Med. 2021 Jul 14;18(7):e1003691. doi: 10.1371/journal.pmed.1003691 (PMC8279302; doi:10.1371/journal.pmed.1003691)
Supplement: S4 Appendix — (PDF) [file pmed.1003691.s010.pdf]

Bergamo, li 2 5 GEN. 2007 Prot. n° U0019644/III.2.4

A.S.L. PROVINCIA DI BERGAMO

Comitato di Bioetica

Segreteria c/o Servizio di Medicina Legale
Via Borgo Palazzo 130 - 24125 BERGAMO
Sistema di Gestione per la Qualità ISO 9001:2000 Certificato CSQ n. 9122
☎ 0352270367 🖷 0352270368

Spett.
Istituto Mario Negri
Centro di Ricerche Cliniche per
le Malattie Rare
ALDO e CELE DACCO'
Villa Camozzi
24020 Ranica (BG)

c.a. Dott.ssa Paola Boccardo

OGGETTO: *Giudizi relativi alla valutazione degli Studi – Comitato di Bioetica della ASL della Provincia di Bergamo del 29 novembre 2006*

Con riferimento a quanto in oggetto indicato, si inviano in allegato i Giudizi espressi dal Comitato di Bioetica della ASL della Provincia di Bergamo, durante la seduta del 29 novembre 2006, sui seguenti Studi:

1) ***Studio VALID "Trial prospettico, randomizzato, multicentrico per valutare se a livelli paragonabili di pressione arteriosa la combinazione ACE inibitore ed antagonista del recettore dell'angiotensina è più efficace dei due farmaci da soli nel ridurre la progressione verso l'insufficienza renale terminale in pazienti diabetici di tipo 2 ad alto rischio e nefropatia conclamata"*** – *Istituto Mario Negri di Bergamo;*

2) ***Studio VARIETY "Trial prospettico, randomizzato, multicentrico per valutare se a livelli paragonabili di pressione arteriosa la combinazione ACE inibitore ed antagonista del recettore dell'angiotensina è più efficace dei due farmaci da soli nel ridurre l'incidenza di microalbuminuria in pazienti diabetici di tipo 2 ipertesi e con valori di albuminuria al limite della normalità"*** – *Istituto Mario Negri di Bergamo.*

Si segnala che è stata valutata anche la Polizza di Assicurazione RC DA SPERIMENTAZINE, stipulata con la Gerling, polizza n. 63/107580/16, decorrenza 14 gennaio 2007 – 14 gennaio 2011, emessa a Milano il 21 dicembre 2006.

Inoltre, si allega qui di seguito, l'elenco dei componenti di questo Comitato di Bioetica, con l'indicazione delle relative qualifiche e dei presenti/assenti alla seduta di cui trattasi:

**Dr. Claudio SILEO (Presidente)**	Direttore Sanitario ASL Bergamo	presente
**Prof. Giancarlo BORRA**	Libero docente in Medicina Legale e delle Assicurazioni	presente
**Dr. Roberto DI SILVESTRE**	Responsabile Servizio di Medicina Legale ASL Bergmao	presente
**Dr. Tiziano GAMBA**	Presidente AVIS Provinciale	presente
**Dr. Francesco LOCATI**	Responsabile USSA Igiene OO.RR. di Bergamo	assente
**Dr.ssa Maria Teresa LORENZI**	Medico di Medicina Generale e Consigliere Ordine dei Medici di Bergamo	presente
**Dr. Franco MAGGIOLO**	Responsabile U.S. Terapia Antivirale OO.RR. di Bergamo	presente
**Dr.ssa Rossana PICCINELLI**	Responsabile U.O. Farmacoeconomia ASL Bergamo	presente
**Inf. Daniela POMINELLI**	Staff Direzione Generale ASL Bergamo	presente
**Dr. Claudio ROZZONI**	Responsabile Centro Bambino e Famiglia ASL Bergamo	presente
**Dr. Giuseppe SAMPIETRO**	Responsabile U.O. Epidemiologia e Sistemi Informativi ASL Bergamo	presente
**Avv. Ettore TACCHINI**	Avvocato e Presidente Ordine degli Avvocati di Bergamo	assente
**Dr. Michele TEDESCHI**	Responsabile Sperimentazione Clinica Istituto Humanitas	presente

Si coglie l'occasione per porgere cordiali saluti.

La Segreteria del Comitato di Bioetica
della ASL della Provincia di Bergamo
Dott.ssa Monica Gussago

All.: come sopra

ASL Provincia di Bergamo
AZIENDA SANITARIA LOCALE

COMITATO DI BIOETICA
DELLA ASL DELLA PROVINCIA DI BERGAMO

Giudizio relativo alla valutazione dello Studio

**TITOLO:**	*TRIAL PROSPETTICO, RANDOMIZZATO, MULTICENTRICO PER VALUTARE SE A LIVELLI PARAGONABILI DI PRESSIONE ARTERIOSA LA COMBINAZIONE ACE INIBITORE ED ANTAGONISTA DEL RECETTORE DELL'ANGIOTENSINA E' PIU' EFFICACE DEI DUE FARMACI DA SOLI NEL RIDURRE L'INCIDENZA DI MICROALBUMINURIA IN PAZIENTI DIABETICI DI TIPO 2 IPERTESI E CON VALORI DI ALBUMINURIA AL LIMITE DELLA NORMALITA'* *A PROSPECTIVE, RANDOMIZED, OPEN LABEL BLINDED EDN POINT (PROBE) TRIAL TO EVALUATE WHETHER, AT COMPARABLE BLOOD PRESSURE CONTROL, COMBINED THERAPY WITH THE ACE INHIBITOR BENAZEPRIL AND THE ANGIOTENSIN II RECEPTOR BLOCKER (ARB) VALSARTAN REDUCES THE INCIDENCE OF MICROALBUMINURIA MORE EFFECTIVELY THAN BENAZEPRIL OR VALSARTAN ALONE IN HYPERTENSIVE PATIENTS TYPE 2 DIABETES AND HIGH-NORMAL ALBUMINURIA*
**PROTOCOLLO:**	VARIETY
**DITTA:**	/
**STRUTTURA di RICERCA:**	*Istituto di Ricerche Farmacologiche Mario Negri di Bergamo*
**SPERIMENTATORE:**	*Dr. Norberto Perico*
**N. Prot. nel Registro delle Sperimentazioni Cliniche:**	44/2006
**N. Prot. in Entrata:**	E0169171/III.2.4 del 3 novembre 2006

Il Comitato di Bioetica della ASL della Provincia di Bergamo in data 29 novembre 2006

Ha esaminato la seguente documentazione:

- Richiesta di autorizzazione e parere unico – Nota dell'Istituto di Ricerche Farmacologiche Mario Negri di Bergamo del 2 novembre 2006;
- Testo del Protocollo di Studio in lingua inglese (versione del 16 ottobre 2006);
- Sinossi del Protocollo di Studio in lingua italiana (versione del 30 ottobre 2006);
- Copia dell'application presentata e approvata dall'AIFA (Proposta 2.3, Cod: FARM5TPY8X);
- Scheda informativa (versione del 30 ottobre 2006);
- Consenso (versione del 30 ottobre 2006);
- Lettera per il medico di medicina generale (versione del 30 ottobre 2006);
- Foglietto illustrativo dei due farmaci utilizzati, da soli o in combinazione, nello Studio, Valsartan e Benazepril;
- Elenco Centro Partecipanti, Centro Coordinatore, Istituto di Ricerche Farmacologiche Mario Negri di Bergamo;
- Domanda di autorizzazione generata dall'OsSC. Codice Eudract: 2006-005954-62;
- Curriculum Vitae dello Sperimentatore responsabile del piano di ricerca, Dr. Norberto Perico;
- Dichiarazione di assunzione di responsabilità - Nota dell'Istituto Mario Negri di Bergamo del 2 novembre 2006;
- Dichiarazione relativa alla comunicazione degli eventi avversi seri o inattesi – Nota dell'Istituto Mario Negri di Bergamo del 2 novembre 2006;
- Fax dell'Istituto di Ricerche Farmacologiche Mario Negri di Bergamo del 20 novembre 2006, unitamente alla copertina dell'OsSC riportante il codice EudraCT: 2006-005954-62;
- Nota dell'Istituto di Ricerche Farmacologiche Mario Negri di Bergamo del 22 novembre 2006;
- Consenso (versione del 28 novembre 2006);
- Consenso al trattamento dei dati sensibili (versione del 28 novembre 2006);
- Testo del Protocollo di Studio in lingua inglese (versione finale del 28 novembre 2006);
- Nota dell'Istituto di Ricerche Farmacologiche Mario Negri di Bergamo del 19 dicembre 2006, unitamente alla versione finale del 15 dicembre 2006 del testo del Protocollo di Studio in lingua inglese.

rilevando che:

lo Studio proposto risulta scientificamente razionale ed eticamente accettabile.

*Si esprime, quindi, **PARERE FAVOREVOLE**, risultando soddisfatti i suddetti prerequisiti etico-scientifici.*

Il Presidente del Comitato di Bioetica
della ASL della Provincia di Bergamo
Dr. Claudio Sileo

Bergamo, lì 2 5 GEN. 2007

Servizio di Medicina Legale

Servizio di Medicina Legale

ISTITUTO DI RICERCHE FARMACOLOGICHE MARIO NEGRI

FONDAZIONE PER RICERCHE ERETTA IN ENTE MORALE CON D.P.R. 361 DEL 5 APRILE 1961 - REG. PERSONE GIUR. PREFETTURA MILANO N. 227, CONTO CORRENTE POST. N. 58337205 COD. FISC. E PARTITA IVA 03254210150 ANAGRAFE NAZIONALE RICERCHE COD. G1690099

Centro di Ricerche Cliniche per le Malattie Rare
ALDO e CELE DACCÒ
Villa Camozzi - 24020 Ranica (Bergamo)
Tel. +39.035.4535.1 - fax +39.035.4535.371
www.villacamozzi.marionegri.it

RECOGNIZED AS A TAX EXEMI ORGANIZATION UNDER SECTION 5 (c) (3) OF THE UNITED STATES (AMERICA INTERNAL REVENUE CODE T/ I.D.No.: 98-6000957
SISTEMA QUALITÀ CERTIFICATO UNI I ISO 9001:2000, ATTIVITÀ DI FORMAZIOI DI NUOVI RICERCATORI IN BIOMEDICIN/

Bergamo, 21 Febbraio 2007

SERVIZIO
DI MEDICINA LEGALE
ARRIV. IL 2 1 FEB. 2007
N° PROT. [illegible]

Gent.mo Dott. Claudio Sileo
Presidente del Comitato di Bioetica
della ASL della Provincia di Bergamo
Via Galliccioli, 4
24121 Bergamo

Oggetto: "A prospective, randomized, open label blinded end point *(probe)* trial to evaluate whether, at comparable blood pressure control, combined therapy with the ACE inhibitor Benazepril and the angiotensin II receptor blocker (ARB) Valsartan, reduces the incidence of microalbuminuria more effectively than Benazepril or Valsartan alone in hypertensive patients with type 2 diabetes and high-normal albuminuria (VARIETY Study)" EUDRACT N° 2006-005954-62, approvato in data 29 novembre 2006.

Gentilissimo Dottor Sileo,

Con la presente Le comunico che il responsabile dello studio in oggetto (supportato dall'Agenzia Italiana del Farmaco - Proposal 2.18, Cod: FARM5TPY8X), come specificato nel contratto stipulato con l'AIFA, sarà il dottor Piero Ruggenenti.

In fede,

Dott. Norberto Perico
Direttore Sanitario

NP/sp
All.

Bergamo, li 2 3 MAR. 2007 Prot. n° U0046903/111.2.1

A.S.L. PROVINCIA DI BERGAMO

Comitato di Bioetica

Segreteria c/o Servizio di Medicina Legale

Via Borgo Palazzo 130 - 24125 BERGAMO

Sistema di Gestione per la Qualità ISO 9001:2000 Certificato CSQ n. 9122

☎ 0352270367 📠 0352270368

Spett.
Istituto Mario Negri
Centro di Ricerche Cliniche per
le Malattie Rare
ALDO e CELE DACCO'
Villa Camozzi
24020 Ranica (BG)

c.a. Dott.ssa Paola Boccardo

OGGETTO: Studio **"VARIETY"** – Vostra nota del 21 febbraio 2007
Responsabile Studio (Dr. Piero Ruggenenti)

In riferimento allo Studio indicato in oggetto, si comunica che nella seduta del 22 marzo 2007 il Comitato di Bioetica della ASL della Provincia di Bergamo ha preso atto della Vostra nota del 21 febbraio 2007.

Ringraziando per la cortese collaborazione, si coglie l'occasione per porgere distinti saluti.

La Segreteria del Comitato di Bioetica
della ASL della Provincia di Bergamo
Dott.ssa Monica [illegible]
