# Supplementary material for: Preventing microalbuminuria with benazepril, valsartan, and benazepril–valsartan combination therapy in diabetic patients with high-normal albuminuria: A prospective, randomized, open-label, blinded endpoint (PROBE) study
Source: PLoS Med. 2021 Jul 14;18(7):e1003691. doi: 10.1371/journal.pmed.1003691 (PMC8279302; doi:10.1371/journal.pmed.1003691)
Supplement: Attachment — Submitted filename: Response to Reviewers.docx [file pmed.1003691.s011.docx]

Comments from the reviewers:

*** Reviewer #1:

Congratulations to the authors and investigators for a well-conducted and clearly presented randomized trial in an area that still is fully elucidated, making the research question important.

**We thank the Reviewer for the rewarding comments on our work.**

Comprehensive manuscript that leaves very few questions or comments:

Suggest explaining the concept of acceleration factors - this is an unfamiliar term.

**As suggested, in the Methods section of the revised manuscript, we provided more detailed information regarding the accelerated failure-time model and the meaning of the acceleration factors (Pages 11-12, lines 254-256; Page 12, lines 262-263).**

Suggest presenting decline in eGFR as annual, not monthly values, for a more clinical comparison.

**As suggested, in the revised version of the manuscript, we presented the slopes of GFR during the study period as annual, instead of monthly, values (Page 17, lines 356-360)**.

Table 1 Lab Parameters (and table S1): The HbA1c values are incorrect, as 5.6% does not correspond to 54 mmol/mol. Suggest to use  <https://urlsand.esvalabs.com/?u=http%3A%2F%2Fwww.ngsp.org%2Fconvert1.asp&e=decac434&h=acf45ee6&f=y&p=y>  to convert between units

**We thank the Reviewer for bringing this point to our attention. In the original version of the manuscript, we reported HbA1c values as both percentage (%) and millimoles per mol (mmol/mol) units with reference to the International Federation of Clinical Chemistry and Laboratory Medicine (IFCC). As the Reviewer properly suggested, in the revised version of Table 1 and S1 Table we expressed HbA1c values as percentage (%) units according to the Diabetes Control and Complications Trial (DCCT), while those reported as millimoles per mol (mmol/mol) units still refer to the IFCC.**

Page line 172: I believe the correct term is "glycated hemoglobin"

**We revised the sentence and removed the term “glycosylated” (Page 10, lines 211-212).**

*** Reviewer #2:

Statistical review

This paper reports a three-arm RCT comparing drugs for preventing microalbuminuria. The trial was conducted with a good length of follow-up using a robust design. The trial was generally reported well although there were some minor differences to the protocol/registration that should be noted - I have listed comments below.

1.        Abstract "The estimated acceleration factors" - this might be clearer if the analysis model being a AFT model is mentioned

**In the revised version of the manuscript we have modified the Abstract, which now reads “Using an accelerated failure-time model, the estimated acceleration factors were 1.410 (95% CI: 0.806 to 2.467, *P*=0.229) for benazepril compared to combination therapy, 0.799 (95% CI: 0.422 to 1.514, *P*=0.492) for benazepril compared to valsartan, and 1.665 (95% CI: 1.007 to 2.746, *P*=0.047) for valsartan compared to combination therapy” (Page 3, lines 56-59).**

2.        Abstract "BP control was similar across groups" - it's not clear why this is reported in the abstract - BP control is not listed as a trial outcome in the ClinicalTrials.gov registration.

**The Reviewer properly pointed that blood pressure (BP) control was not one of the study outcomes. However, it was a major treatment target. Indeed, in the Introduction section of the original manuscript we emphasized that we wanted to test the effects of different treatment regimens on microalbuminuria and other outcomes at comparable BP control. Thus, in all treatment groups treatment was titrated in order to achieve the same BP target. This is because BP reduction, independent of the treatment used to reduce BP, is per se a determinant of albuminuria reduction. Conversely, results of most previous studies showing that dual RAS blockade combining an ACE inhibitor and an ARB may reduce albuminuria more effectively than single blockade with ACE inhibitor or ARB monotherapy in subjects with or without diabetes (Mogensen CE et al. BMJ 2000; 321:1440-1444; Fried LF et al. N Engl J Med 2013; 369:1892-1903; Rossing K et al. Diabetes Care 2002; 25:95-100; Jacobsen P et al. J Am Soc Nephrol 2003; 14:992-999), were largely flawed by larger BP reduction achieved with combination therapy than with monotherapy (Mogensen CE et al. BMJ 2000; 321:1440-1444; Fried LF et al. N Engl J Med 2013; 369:1892-1903; Rossing K et al. Diabetes Care 2002; 25:95-100; Jacobsen P et al. J Am Soc Nephrol 2003; 14:992-999). Thus, whether the superior antialbuminuric effect of combination therapy was mediated by more effective inhibition of the RAS or rather by larger BP decline *per se,* remained elusive. This is an issue with major practical implications because, in the case of a BP-driven effect, any combined therapy even with medications that do not directly interfere with the RAS, would have stronger antiproteinuric effects than ACE inhibitor or ARB monotherapy (Page 20, lines 399-402; Page 20, lines 406-408; Page 21, lines 409-412 of the original version of the manuscript). Therefore, our present study provides the first evidence that ACE inhibitor plus ARB dual therapy and either agent alone at equivalent antihypertensive doses and comparable BP control are similarly effective in the primary prevention of microalbuminuria in patients with type 2 diabetes and hypertension.**

**In light of the above considerations, we would like to keep the information that BP control was similar across groups in the revised version of the Abstract.**

3.        Randomization and masking - I note the protocol mentions randomisation was stratified by centre, which is not stated here. Also adding the possible block sizes that were randomly chosen would be useful.

**We thank the Reviewer for highlighting this important aspect. We confirmed that the randomization was stratified by centre. This information has been now added in the new version of the manuscript (Page 9, lines 182-182). Details on block sizes have been specified (Page 9, line 187).**

4.        Outcome measures: The cardiovascular events outcome was not listed on the registration page or in the protocol from what I can see. I would recommend it's made clear that this was no a pre-specified outcome.

**According to the Study Protocol (Page 10) one of the secondary endpoints of the VARIETY trial was the incidence of major cardiovascular events, defined as sudden death and fatal or non-fatal stroke and acute myocardial infarction. However, in the original version of the manuscript, we used a broader definition of major adverse cardiovascular events, which - in addition to sudden cardiac death and fatal or non-fatal stroke and acute myocardial infarction – included also coronary or peripheral revascularization, and amputation because of critically ischemic limb. However, for consistency with the study protocol, in the revised version of the manuscript, we decided to focus on the combined endpoint of sudden death and fatal and non-fatal acute myocardial infarction or stroke (novel Figure 4; Page 17, lines 359-362). Moreover, in the revised version of Table 2, which outlines the number of patients with at least one serious adverse event during the study period, peripheral artery disease/revascularization, peripheral artery disease/amputation, and coronary revascularization have been classified as “Other cardiovascular events” instead of “Major cardiovascular events”.**

**Thus, in the revised Methods section, we clarified that treatment effects on the composite cardiovascular outcome of sudden cardiac death, acute myocardial infarction, stroke, coronary or peripheral revascularization, or amputation because of critically ischemic limb were evaluated in non-prespecified, exploratory analyses (Page 10, lines 224-225; Page 12, lines 274-277). Consistently, the Figure showing the results of these analyses has been moved to the Supporting Information (S2 Figure of the revised manuscript).**

5.        Sample size and statistical analysis: the authors mention 'based on the interim analysis' - can more information be provided about this? Was the sample size formally reassessed in this trial, and if so was it based on blinded or unblinded information?

**We agree with the Reviewer that the mentioned sentence is unclear. Actually, there was no formal sample size reassessment on the basis of interim, unblinded results. The point is that, upon request of Agenzia Italiana del Farmaco (AIFA) – that is: the Italian Agency of Medicines -, we have updated the sample size using data from the interim analysis in aggregated, blinded fashion. This aspect has now been clarified in the statistical methods of the revised version of the manuscript (Page 11, lines 234-237).**

6.        Sample size and statistical analysis - I did not see where the valsartan monotherapy arm came into the analysis - the first line of this section implies this wasn't a primary analysis. If it was, the authors could comment on multiple testing, which is mentioned in the protocol but not in the paper.

**In the revised version of the manuscript, we clarified that secondary objectives involved comparisons of combination therapy versus valsartan monotherapy, and benazepril monotherapy versus valsartan monotherapy for new onset of persistent microalbuminuria. We also specified that these descriptive comparisons were carried out by using the same methodological approach used for the primary comparison between benazepril monotherapy and combination therapy, but at the 2.5% level of significance (two-sided test) in order to take into account for the multiple testing issue (Page 12, lines 268-273). This issue has been clarified in the statistical methods of the revised manuscript.**

7.        Line 221 - I did not follow how the GFR slope was modelled - was difference between arms estimated? If so it is not reported in the results (just the estimated slope in each arm separately).

**In the revised version of the Methods section, we specified that a single-slope linear model was used and that between group differences in GFR slopes were assessed by Wilcoxon rank sum test (Page 12, lines 277-280). In the original version of the Results section, we reported that the monthly rate of GFR decline was similar on benazepril (0.14 [0.04 to 0.34] mL/min/1.73 m^2^), combination therapy (0.22 [0.06 to 0.30] mL/min/1.73 m^2^) or valsartan (0.12 [-0.08 to 0.24] mL/min/1.73 m^2^) (Page 14, lines 298-300 of the original version of the manuscript). For the sake of clarity, in the revised manuscript we specified that the rate of GFR decline (expressed as annual, instead of monthly, value as per Reviewer #1 suggestion) did not differ significantly among the benazepril (1.73 [0.44 to 4.13] mL/min/1.73 m^2^), combination therapy (2.61 [0.77 to 3.59] mL/min/1.73 m^2^) and valsartan (1.45 [-0.94 to 2.92] mL/min/1.73 m^2^) groups (Page 17, lines 356-360).**

8.        BP and glycemic control - please report these results with estimated differences between arms and CIs rather than just p-values.

**In the revised version of the manuscript we reported, for systolic blood pressure, diastolic blood pressure and HbA1c levels, mean differences between treatment arms during the study period along with 95% confidence intervals and *P* values (Page 19, lines 389-398). To avoid data redundancy we deleted *P* values the related figure.**

James Wason

*** Reviewer #3:

This manuscript reports the results of a well-designed and rigorously conducted RCT of benazepril vs. valsartan vs. combination therapy (at half dose) in participants with type 2 diabetes to evaluate the impact on the incidence of microalbuminuria. The manuscript is very well written and the data are presented clearly. The issue investigated is clinically relevant and the conclusions are clear and definitive - no benefit was observed with combination versus monotherapy. In addition to answering this primary research question, the results provide further evidence that ACE inhibitor therapy affords similar benefit to angiotensin receptor blocker therapy.

**We thank the reviewer for the rewarding considerations on our work.**

Comments:

*        Page 5, rows 109-110: This sentence is a little unclear and could be shortened to: "Secondarily, we compared the protective effect against microalbuminuria development of two monotherapies in the same population".

**In the revised version of the manuscript, we have shortened the sentence as suggested (Page 7, lines 151-152).**

*        Page 10, rows 216-217: The authors should provide a more detailed explanation of "acceleration factors" since these are not commonly reported.

**As suggested, in the Methods section of the revised manuscript, we provided more detailed information regarding the accelerated failure-time model and the meaning of the acceleration factors (Pages 11-12, lines 254-256; Page 12, lines 262-263).**

*        Table 1: A footnote states that measured GFR was obtained in a subgroup of participants. It would be helpful also to provide the number (n=77)

**As suggested, we have added the number of patients in whom GFR was measured by iohexol plasma clearance in the footnote of Table 1.**

*        Measured GFR (plasma iohexol clearance) was assessed in only a minority of participants but serum creatinine was measured in all. The authors should therefore report estimated GFR values for all participants in Table 1. Also, estimated GFR could be used to calculate the slope of GFR over time in all participants.
**As per Reviewer’s suggestion, in Table 1 of the revised** **manuscript we added GFR estimated by the** **Chronic Kidney Disease Epidemiology Collaboration (CKD-EPI) equation (Levey AS et al. Ann Int Med 2009; 150:604-12) at baseline in the three treatment groups (Page 10, lines 212-214). We also calculated the slopes of estimated GFR during the follow-up in the overall study population and according to randomization group. However, we would prefer not to report these data because, as we showed and discussed in several previous studies and reviews (the Reviewer may wish to consider just as an example: Gaspari F et al. Kidney Int 2013; 84:164-173; Porrini E et al. Nat Rev Nephrol 2019; 15:177-190), serum creatinine-based estimation formulas fail to provide reliable estimates of GFR changes over time, especially in patients with type 2 diabetes mellitus without overt nephropathy. This decision is consistent with all our previous works and is aimed to avoid providing potentially misleading information to the readers. We are confident the Reviewer will understand our position.**

***
