# Supplementary material for: Preventing microalbuminuria with benazepril, valsartan, and benazepril–valsartan combination therapy in diabetic patients with high-normal albuminuria: A prospective, randomized, open-label, blinded endpoint (PROBE) study
Source: PLoS Med. 2021 Jul 14;18(7):e1003691. doi: 10.1371/journal.pmed.1003691 (PMC8279302; doi:10.1371/journal.pmed.1003691)
Supplement: Attachment — Submitted filename: Response to Reviewers.docx [file pmed.1003691.s012.docx]

Requests from Editors:

Please remove the time restraints from your data statement (submission form) - so as to comply with PLOS' data policy, data must be available in principle from the date of publication.

**As suggested, we have removed time restrains from our data statement.**

At lines 52 and 171 there is a slight difference in the way the participating units are described.

**Thank you for bringing to our attention this inconsistency, that we have addressed in the revised version of the manuscript (Page 3, lines 52; Page 8, line 171).**

At line 294, please make that "Two hundred ...".

**We have changed “Two-hundreds” in “Two hundred” (Page 14, line 294).**

Throughout the text, please use the style "... 3 study groups ...", although numbers should be spelt out at the start of sentences.

**We have revised the text as suggested**.

Currently, the Acknowledgements section appears twice at the end of the ms.

**The Acknowledgements section now appears once** **at the end of the revised version of the manuscript.**

Comments from Academic Editor:

In my view the authors have responded adequately to all reviewer comments. This is an important paper as it represents another (and possibly the final) milestone in the long-running exploration of whether inhibition of the renin-angiotensin system with ACE inhibitors or angiotensin receptor blockers or their combination offers superior renoprotective benefits (see Taal MW and Brenner BM. Renoprotective benefits of RAS inhibition: from ACEI to angiotensin II antagonists. Kidney Int. 2000 May;57(5):1803-17). There was a compelling rationale for combining ACE inhibitor and angiotensin receptor blocker therapy but clinical trials have not shown renoprotective benefit in different types of kidney disease.

**We thank the Academic Editor for the rewarding comments on our work.**

I agree with the authors that comparison of blood pressure control between groups should be reported in the abstract as this is vital for interpretation of the results.

**Thank you for your comment. We have kept in the abstract the information that blood pressure control was similar across groups.**

There are a few very minor language issues that could be resolved during production or during a minor revision. I therefore recommend minor revision with a view to accepting this paper for publication.

**As suggested, in the revised version of the manuscript we have addressed the highlighted minor language issues.**

Comments:
Line 82-83: suggest rephrase to “Treatment effect was, however, associated with greater blood pressure reduction.”

**We have rephrased the sentence as suggested (Page 4, lines 76-77).**

Line 162-163: suggest rephrase to “This was a phase 3, multicenter, parallel PROBE trial [14], that included male and female patients aged over 40 years with type 2 diabetes (WHO criteria) and a known history of diabetes not exceeding 25 years.”

**We have rephrased the sentence accordingly (Page 8, lines 157-159).**

Line 172: word missing, suggest change to “…excluded patients with drug or alcohol abuse…”

**We have revised the sentence as suggested (Page 8, line 167).**

Line 243: suggest change to “…who was not directly involved in conducting the study.”

**We have revised the sentence as suggested** **(Page 9, line 184).**

Line 360-362: suggest change to “Non-pre-specified analyses considered in addition to MACE, included coronary or peripheral revascularization or amputation because of critically ischemic limb.”

**We have modified the sentence as suggested (Page 10, lines 222-224).**
